# Analysis of the microbial diversity in the fecal material of the critically endangered orangutan.

**DOI:** 10.17912/micropub.biology.001554

**Published:** 2025-03-28

**Authors:** Carly M. Smith, Dan Powell, Xiaoling Wan, JinSong Zheng, Durward L. Bevis, Richard W. McLaughlin

**Affiliations:** 1 Gateway Technical College, Kenosha, Wisconsin, United States; 2 Racine Zoo, Racine Wisconsin, USA; 3 Wuhan Polytechnic University, Wuhan, Hubei, China; 4 Chinese Academy of Sciences, Wuhan, China

## Abstract

The orangutan ( *Pongo* spp.) is a critically endangered species. Today, populations of these animals are rapidly declining by up to 75%. They are found in the tropical rainforests of Borneo and Sumatra. In this study, using a next generation sequencing approach, the bacterial and fungal diversity in the fecal material of orangutans living in the Racine Zoo were investigated. The most predominant bacterial phyla were the Bacillota along with Bacteroidota. The most predominant fungal phylum was Ascomycota. Finally, the various functions of the bacterial communities present in the fecal material were predicted with PICRUSt2 using the KEGG database.

**Table 1. Bacterial and fungal phyla present in the fecal material of the orangutan along with predicted cellular processes:** An analysis of the bacterial (1A) and fungal diversity (1B) at the phyla level in the fecal material of the orangutan. The different functions of the bacterial communities present in the fecal material were also predicted with PICRUSt2 using the KEGG database (1C). * relative percentage of operational taxonomic units (OTUs).

**Table:** 

Predicted Phyla and Function	Orangutan 1	Orangutan 2
<b>1A. Bacteria Phyla</b>	<b>Jenny*</b>	<b>Max*</b>
Actinobacteriota	4.202	2.897
Bacteroidota	21.068	20.576
Campilobacterota	0.242	0.997
Cyanobacteriota	0.051	0.031
Desulfobacterota	0.264	0.225
Fibrobacterota	0.068	0
Bacillota	67.297	65.106
Fusobacteriota	0	0.004
Patescibacteria	0	0.056
Planctomycetota	0.056	0.056
Pseudomonadota	0.783	1.744
Spirochaetota	2.053	1.627
Synergistota	0.02	0.205
Verrucomicrobiota	0.181	1.581
<b>1B. Fungi Phyla</b>	<b>Jenny*</b>	<b>Max*</b>
Ascomycota	91.05	64.66
Basidiomycota	7.01	3.06
Mucoromycota	0	14.59
<b>1C. Predicted Biological Function</b>	<b>Jenny</b>	<b>Max</b>
Cellular Processes	4.09%	4.36%
Environmental Information Processing	5.28%	5.41%
Genetic Information Processing	8.75%	8.91%
Human Diseases	3.14%	3.12%
Metabolism	76.99%	76.46%
Organismal Systems	1.74%	1.74%

## Description

Approximately 12,500 years ago during the Pleistocene, orangutans ( *Pongo* spp.) were found throughout Southeast Asia. Their territory ranged from Southern China in the north to Java in the south (Chapman et al., 2020; van Schaik et al., 2001). Unfortunately, there has been a severe population decline. Because of greater global demands for products, such as sugar cane, rice, oil palm, natural rubber, and tropical hardwoods, rapid and widespread deforestation has occurred (Laurance et al., 2014). This has greatly impacted many animals including the orangutan (Nantha and Tisdell, 2009; Wich et al., 2014). Today they are only found in the tropical rainforests of Borneo and Sumatra (Chapman et al., 2020). *Pongo abelii* live on Sumatran Island and *P. pygmaeus* on Borneo (Ancrenaz et al., 2017). Unfortunately, both species are currently listed in the IUCN Red Data Book as critically endangered (IUCN, 2016, 2017).

Because the microbial diversity is critical in maintaining health (Wu et al., 2016), the emphasis of this research was to examine both the bacterial and fungal diversity present in the fecal material of orangutans. A better understanding of their gastrointestinal flora could positively impact the health of these animals, which may aid in their preservation.

In the present study, the fecal material from two adult orangutans were analyzed. A total of 1, 052, 448 and 1, 141, 876 quality sequences were generated for Jenny and Max, respectively. The number of operational taxonomic units (OTUs) were 251 and 259, the Pielou's Evenness index was 0.87 and 0.86 and the Shannon diversity index was 6.95 and 6.88 for Jenny and Max, respectively. Ley et al., (2008) showed that the fecal material of carnivorous animals had less bacterial diversity compared to herbivores.

The fungal diversity in animals that are not used for food production has rarely been determined. However, in animals, such as whales (Guass et al., 2016), giraffes (Schmidt et al., 2018), and East Asian finless porpoises (Wan et al., 2018) this has been examined. In this study, a total of 278, 734 and 303, 678 quality sequences were generated for samples Jenny and Max, respectively. The number of OTUs were 19 and 15, the Pielou's Evenness index was 0.52 and 0.79 and the Shannon diversity index was 2.19 and 3.10 for samples Jenny and Max, respectively.

The predominant bacterial phylum was Bacillota (synonym Firmicutes) in samples Jenny and Max, containing 67.297% and 65.106% of the OTUs, respectively ( Figure 1A ). As well, a high abundance of Bacteroidota (synonym Bacteroidetes) 21.068% and 20.576% of the OTUs was also detected in Jenny and Max, respectively ( Figure 1A ). In a recent study, Bacillota and Bacteroidota were the two dominant phyla in the non-human primates (NHPs) Siamangs, white-handed gibbons, and Bornean orangutans (Ying et al., 2022). Bacillota was the largest portion of the sequences, followed by Bacteroidetes of a member of the genus *Colobus* (Yildirim et al., 2010). It is common to detect a high abundance of Bacillota followed by Bacteroidota in the gut of terrestrial mammals, apart from terrestrial carnivores (Nelson et al., 2013). In our study, a similar trend was found. One reason for this finding is that Bacillota have the potential to degrade lignin (Liu et al., 2019; Que et al., 2022) and Bacteroidota has genes which encode enzymes to hydrolyze complicated plant polysaccharides (Grondin et al., 2017).

In our study, the fungal microbiome was also characterized. The predominant fungal phylum was Ascomycota for both orangutans ( Figure 1B ). Ascomycota has been found in the fecal material of Blue whales (Guass et al., 2016), a Caucasian woman (Gouba et al., 2013), insects (Kurtzman and Robnett, 2012), giant pandas (Tun et al., 2014), dogs (Foster et al., 2013; Handl et al., 2011), cats (Handl et al., 2011), and yaks (Li et al., 2022). In a recent study, Ascomycota was the most predominant phylum from three to six months of age in yaks (Li et al., 2022). The gut mycobiota of Tibetan macaques was dominated by Ascomycota and Basidiomycota (Sun et al., 2018). However, overall little is known about the fungi present in the gut of NHPs (Sun et al., 2020).

PICRUSt2 was used to predict the bacterial functional potential based on the KEGG database. At pathway level 1, metabolic pathways were the most abundant, accounting for 76.99 and 76.46% of the identified pathways in samples Jenny and Max, respectively. This was followed by genetic information processing, environmental information processing, cellular processes, human diseases, and organismal systems ( Figure 1C ).

## Methods

Ethics statement

IACUC approval was granted by the Racine Zoo for the collection of fecal samples.

Animals and sample collection

The two orangutans, living in the Racine Zoo, Racine WI, used in this study were Max, Male, DOB 03/06/1986, and Jenny, Female, DOB 05/15/1985. Both were born in the Henry Villas Zoo in Madison WI. They are a hybrid of Sumatran and Borneo species. Their diets consisted primarily of a variety of commercially prepared primate "biscuits" such as Marion Leafeater, plus a variety of fruits and vegetables. The fecal material from the orangutans, one sample/animal, were collected in September 2021 and were placed into separate containers. The two fecal samples were placed in a −40 °C freezer and stored until used for laboratory experiments.

Bacterial and fungal diversity

The inner portions of the orangutan fecal samples were sent to the SeqCenter ( https://www.seqcenter.com/ ). DNA was isolated using the Quick-16S kit (Zymo Research, Irvine, USA). To determine the bacterial diversity phased primers targeting the V3/V4 regions were used. The primer sequences were forward 5′-CCT ACG GGD GGC WGC AG-3′, 5′-CCT AYG GGG YGC WGC AG-3′ and reverse 5′-GAC TAC HVG GGT ATC TAA TCC-3′. After clean-up and normalization sequencing was done on a P1 600 cyc NextSeq 2000 Flow cell (Illumina, San Diego, USA) in which 2x301bp PE reads were generated. Both quality control and adapter trimming were performed using BCL Convert v4.0.3. ( https://emea.support.illumina.com/sequencing/sequencing_software/bcl-convert.html ). To process the sequences QIIME 2 v2021.11 was used (Bolyen et al., 2019). Primer sequences were removed using the cutadapt plugin (Martin, 2011) and sequences were denoised using the dada2 plugin (Callahan et al., 2016). Operational taxonomic units (OTUs) were assigned using the Silva 138 99% OTUs full-length sequence database and VSEARCH (Rognes et al., 2016). OTUs were collapsed to the lowest taxonomic units. Counts were then converted to reflect relative frequency.

The same methods were used to determine the fungal diversity using phased primers targeting the ITS2 region which were forward 5′- GCA TCG ATG AAG AAC GCA GC-3′ and reverse 5′- TCC TCC GCT TAT TGA TAT GC-3′. OTUs were assigned using the Unite 8 99% OTUs full-length sequence database and VSEARCH (Rognes et al., 2016).

Default settings were used for all programs and plugins.

Functional prediction

All the representative sequences of OTUs and the rarefied OTU abundance table from 16S rRNA gene sequences were used to predict microbial function through PICRUSt2 (Phylogenetic Investigation of Communities by Reconstruction of Unobserved States) (Douglas et al., 2020), using QIIME2 (Bolyen et al., 2019). Gene families were mapped against the KEGG database for bacteria.

Nucleotide sequence accession numbers

The NGS data have been deposited in the Sequence Read Archive (SRA) under accession numbers SRR24116963 to SRR24116966.
